# Solitary adrenal metastasis from salivary duct carcinoma of the parotid gland successfully treated by surgery: A case report

**DOI:** 10.1097/MD.0000000000024011

**Published:** 2021-01-15

**Authors:** Yusuke Amano, Kentaro Tsuji, Atshushi Kihara, Daisuke Matsubara, Noriyoshi Fukushima, Hiroshi Nishino, Toshiro Niki

**Affiliations:** a Department of Pathology; b Department of Otolaryngology, Jichi Medical University, Yakushiji, Shimotsuke, Tochigi, Japan.

**Keywords:** salivary duct carcinoma, salivary gland, adrenal gland, solitary metastasis

## Abstract

**Rationale:**

Salivary duct carcinoma (SDC) is a rare and highly aggressive cancer with a poor prognosis. SDC demonstrates a potential for invasive growth with early regional and distant metastasis to organs, such as bone, lung, liver, and brain. Because, adrenal gland metastasis from SDC is rare, its treatment options are not well established. Herein, we report a case of SDC metastasis from the parotid gland to the adrenal gland, which was successfully treated by surgery.

**Patient concerns:**

The patient had an abnormal but painless lump on the right parotid gland. The size of the mass had increased over a period of 3 years. The patient underwent complete removal of the right parotid gland and radical neck dissection followed by adjuvant radiotherapy and chemotherapy. Two years later, a mass was identified in the left adrenal gland by computed tomography. As no local recurrence or metastasis to other organs was observed, the patient underwent adrenalectomy.

**Diagnoses:**

Metastasis of SDC in the adrenal gland was confirmed by histopathological examination of the adrenalectomized specimen.

**Interventions:**

After adrenalectomy, the patient was followed-up without adjuvant therapy.

**Outcomes:**

The patient was well and alive during the 13-month postoperative follow-up period without any complications.

**Lessons:**

Surgical resection of solitary metastatic lesion may show a survival benefit with metastatic SDC.

## 1 Introduction

Salivary duct carcinoma (SDC) is a rare and aggressive salivary gland malignancy that was first described by Kleinsasser et al in 1968^[1]^. Histologically, SDC is a high-grade adenocarcinoma with features similar to that of breast ductal carcinoma such as duct formation, solid, papillary, cribriform pattern, and intraductal comedo necrosis.^[2]^ SDC predominantly originates from the parotid gland, followed by the submandibular gland and minor salivary glands. Patients with SDC often present with lymph node metastasis at diagnosis; therefore, extensive surgical resection followed by adjuvant radiotherapy is generally indicated.^[3]^ Furthermore, SDC frequently metastasizes to the bone, lung, liver, and brain.^[4,5]^ However, metastasis to the adrenal gland is very rare. Herein, we report a 63-year-old man with SDC that had metastasized to the adrenal gland.

## 2 Case presentation

A 63-year-old male discovered an abnormal but painless lump on the right parotid gland. The size of the mass had increased over a period of 3 years. The patient was admitted to our hospital for magnetic resonance imaging of the jaw and face, which revealed a mass (3 cm) in the right submandibular gland.

The patient underwent fine-needle aspiration of the right subparotid gland mass, and cytological examination confirmed a diagnosis of SDC. Subsequently, complete removal of the right submandibular gland and radical neck dissection were performed.

**Figure 1:**
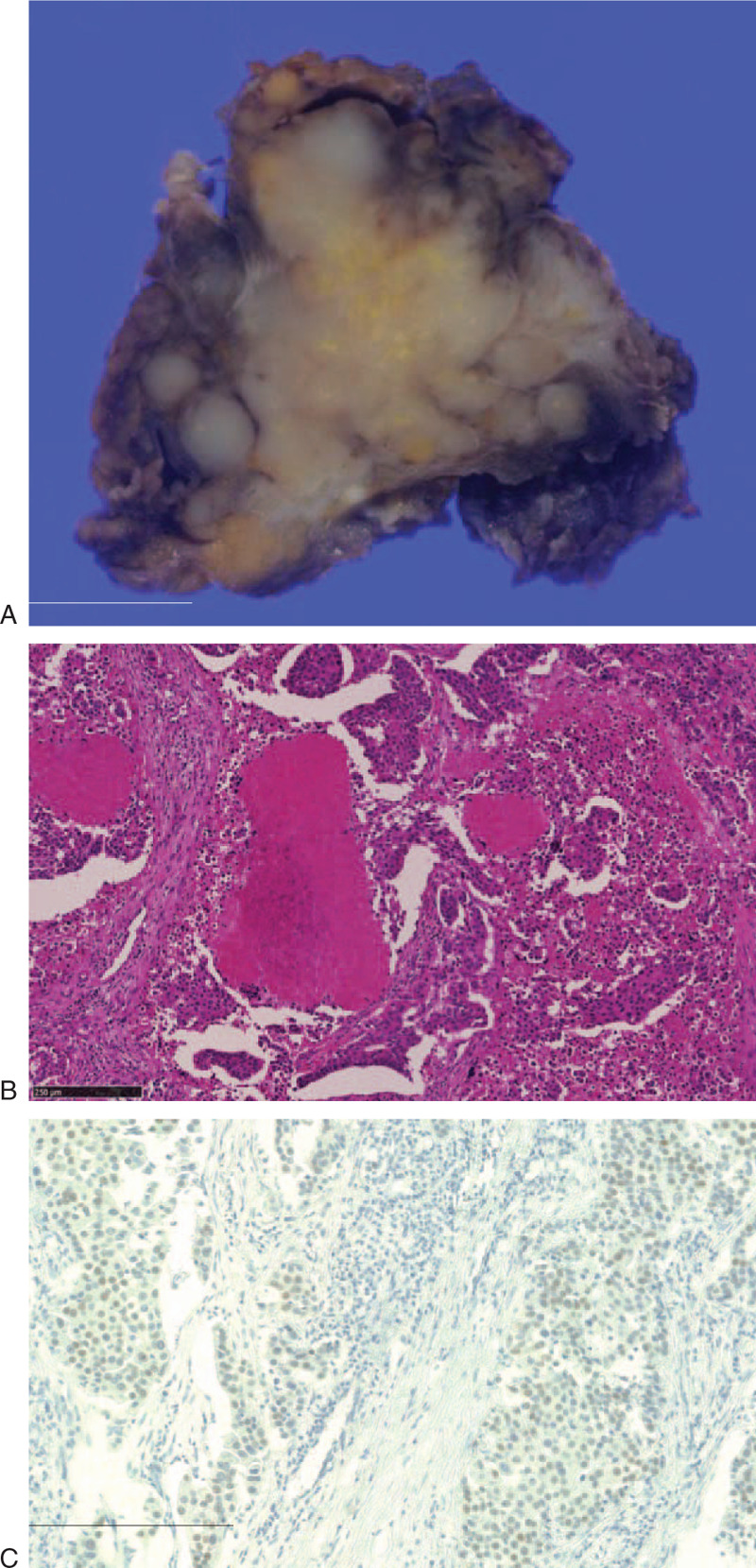
Macroscopic and microscopic findings of salivary duct carcinoma (SDC). Cut surface of the resected specimen showed a whitish-yellow colored mass (A; Bar, 1 cm). SDC with roman bridges and foci of comedo necrosis. Hematoxylin & Eosin (H&E) staining (B; Bar, 250 μm). Weak nuclear expression of androgen receptor in SDC (C; Bar, 250 μm).

**Figure 2:**
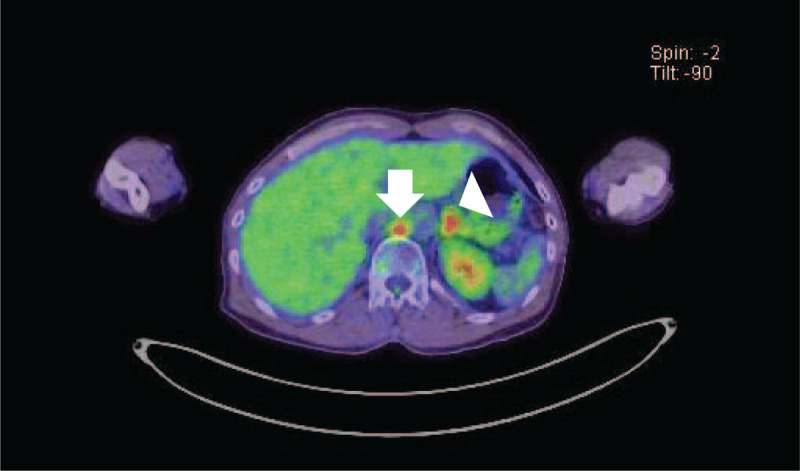
Positron emission tomography and computed tomography showing enlarged adrenal gland (arrowhead) and para-aorta lymph node (arrows).

Macroscopically, the surgical specimen demonstrated the features of a solid tumor with a light brown tone and the presence of a white- or yellow-colored area (Fig. 1A). Microscopically, the tumor consisted of an atypical ductal component with the roman bridges and foci of comedo necrosis (Fig. 1B). No sarcomatoid or mucin-producing components were detected. Immunohistochemically, the tumor cells were weakly positive for androgen receptor (Fig. 1C) and negative for human epidermal growth factor receptor 2. Lymphovascular, perineural, and adjacent structure invasion along with cervical lymph node metastasis (2 of 21) were also observed. These histological findings indicated a diagnosis of SDC (pathological staging, pT3N2bM0). The patient received adjuvant radiotherapy (total 70 Gy) and chemotherapy (3 courses of cisplatin for 3 weeks) after surgery. Two years after the initial operation, PET (positron emission tomography) and CT (computed tomography) revealed enlargement of the left adrenal gland and the para-aortic lymph nodes (Fig. 2). No local recurrence or metastasis to other organs was observed. The patient was diagnosed with a metastatic malignancy and underwent adrenalectomy.

**Figure 3:**
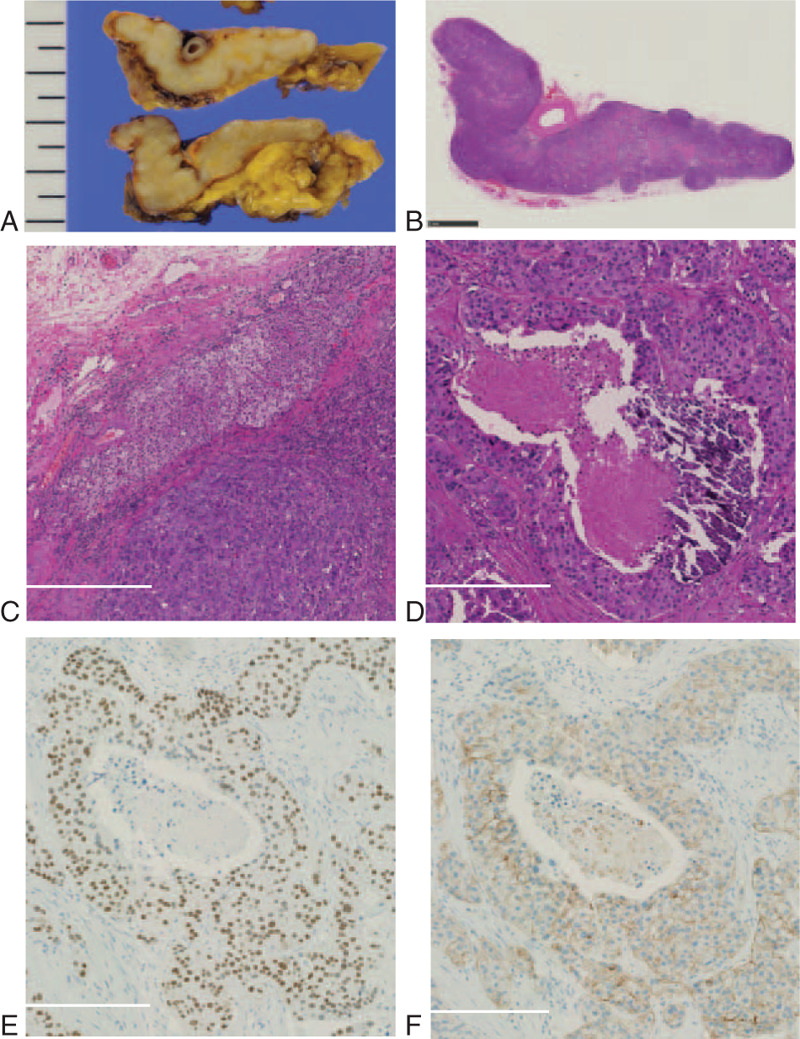
Macroscopic and microscopic findings of salivary duct carcinoma (SDC) in adrenal gland. Cut surface of the resected specimen was replaced with a whitish-yellow colored mass (A). Semi-macroscopically, adrenal gland was mostly replaced with SDC (B; Bar, 5 mm). Scant amounts of adrenal gland were seen adjacent to SDC, H&E staining (C; Bar, 500 μm). High-power field finding of SDC, H&E staining (D; Bar, 500 μm). Nuclear expression of androgen receptor in SDC (E; Bar, 500 μm). Membranous expression of human epidermal growth factor receptor2 in SDC (F; Bar, 500 μm).

Macroscopically, the left adrenal gland was mostly replaced with a whitish-yellow mass (Fig. 3A). Semi-macroscopically, the left adrenal gland was mostly replaced with tumor (Fig. 3B). Microscopically, scant amounts of adrenal gland component were observed adjacent to SDC (Fig. 3C). SDC had the roman bridges and foci of comedo necrosis (Fig. 3D).

Immunohistochemically, the tumor cells were positive for androgen receptor (Fig. 3E) and human epidermal growth factor receptor2 (Fig. 3F). Para-aortic lymph node metastasis was noted (2 of 11). The patient was diagnosed with metastatic SDC in the adrenal gland. He recovered well after the surgery, and no recurrence was noted postoperatively during the 13-month follow-up period.

## 3 Discussion

Metastasis to the adrenal gland is extremely rare and usually discovered at autopsy.^[6,7]^ The most common malignant neoplasms that metastasize to the adrenal gland are lung cancer, breast cancer, colon cancer, renal cell carcinoma, and malignant melanoma.^[6–8]^ Distant metastasis of SDC has been reported in 37.5% to 82% of cases and generally involves the lung, liver, bone, and brain.^[3,4]^ Studies have reported metastasis to the bone marrow, breast, stomach, and gingiva.^[9–12]^ However, there are no reports on the metastasis of SDC to the adrenal gland.

The majority of patients with adrenal gland metastasis are asymptomatic and do not complain of abdominal pain or adrenal insufficiency.^[13]^ About 80% to 90% of the adrenal gland must be replaced or destroyed by the tumor cells prior to adrenal insufficiency.^[13]^ Primary adrenal gland malignant tumors, such as adrenocortical carcinoma, usually secrete excessive amounts of hormones; in some cases, they may be associated with hemorrhage, abdominal distention, or discomfort.^[13,14]^ Nevertheless, it is difficult to distinguish metastatic adrenal carcinoma from primary adrenal tumor, which can lead to misdiagnosis.^[15]^

Solitary adrenal metastasis of SDC is extremely unusual, but adrenal metastasis in the presence of multiple synchronous metastases to other organs during the terminal stages of cancer is not an uncommon finding.^[15–17]^ Although the incidence of adrenal gland incidentalomas in patients with colorectal cancer was reported to be 10.5%, the incidence of adrenal metastasis with widespread systemic disease was 0.4%, whereas no cases of solitary adrenal metastasis were noted.^[18]^

The spread of adrenal metastasis occurs by arterial, portal venous, or lymphatic routes.^[19]^ Generally, hematogenous spread is considered as the major route of metastasis of the primary carcinoma to the adrenal gland.^[19]^ Katayama et al suggested a plausible route for hematogenous metastasis from the primary site to the adrenal gland via the lung.^[20]^

Serum carcinoembryonic antigen levels are useful indicators of adrenal gland metastasis after colorectomy, which can be confirmed by imaging modalities such as CT, magnetic resonance imaging, and PET-CT. Other indicators of metastasis by CT and PET-CT include a mass >2 cm in size, a mean attenuation of >10 HU, and a maximum standardized uptake value (max) of >3.1.^[18,19,21]^ The findings in the present case (diameter, 2.5 cm; standardized uptake value max, 7.30) were consistent with a previous study.

In colorectal and non-small lung cancer patients with distant metastasis, there was a marked difference in prognosis between the cases with single organ (solitary) metastasis and those with multi-organ metastases.^[22,23]^ There are case reports indicating that surgical resection of single adrenal metastasis of colorectal cancer may be beneficial for patient survival.^[16,20]^ Also, studies have shown that curative resection of single organ metastasis provides a survival benefit for cancer of the esophagus and breast.^[24,25]^ Whether metastasectomy of a single organ metastasis shows survival benefit for head and neck and salivary gland cancer remains controversial.^[26,27]^

We present a very rare case of a 63-year-old man with single-organ SDC metastasis to the adrenal gland. Our case indicates that curative resection of solitary adrenal metastasis may provide survival benefit as reported for salivary gland cancer patients and other types of cancer patients as well. Further studies are needed to clarify this issue.

## Author contributions

**Conceptualization:** Yusuke Amano and Toshiro Niki.

**Data curation:** Yusuke Amano, Kentaro Tsuji, Atshushi Kihara, Daisuke Matsubara,

**Formal analysis:** Yusuke Amano, Kentaro Tsuji, Atshushi Kihara, Daisuke Matsubara,

**Investigation:** Yusuke Amano, Kentaro Tsuji, Atshushi Kihara, Daisuke Matsubara, and Noriyoshi Fukushima.

**Methodology:** Yusuke Amano and Toshiro Niki, Noriyoshi Fukushima, and Hiroshi Nishino.

**Project administration:** Yusuke Amano.

**Resources:** Yusuke Amano, Kentaro Tsuji, Atshushi Kihara, Daisuke Matsubara.

**Software:** Yusuke Amano.

**Supervision:** Toshiro Niki.

**Validation:** Yusuke Amano, Kentaro Tsuji, Atshushi Kihara, Daisuke Matsubara.

**Visualization:** Yusuke Amano.

**Writing – original draft:** Yusuke Amano.

**Writing – review & editing:** Yusuke Amano and Toshiro Niki.

## Abbreviations

Abbreviations: CT = computed tomography, PET = positron emission tomography, SDC = salivary duct carcinoma.
